# The Transactions of the Provincial Medical and Surgical Association

**Published:** 1840

**Authors:** 


### The Transactions of the Provincial Medical and Surgical Association. Instituted 1832. Volume VIII. London: J. Churchill, 1839.

The contents of the present volume, in the publication of which an unavoidable delay has taken place, are as follows:—Proceedings of the Association at Liverpool; Report of the Council; Report on Medical Reform; Report of Benevolent Fund; Report on Quackery; Report of the Section appointed to enquire into the present state of Vaccination, as read at the Anniversary Meeting of the Provincial Medical and Surgical Association, held at Liverpool, July 25, 1839; The Retrospective Address delivered at the Seventh Anniversary Meeting of the Provincial Medical and Surgical Association, held at Liverpool, July 24th and 25th, 1839, by John Addington Symonds, M. D., Senior Physician to the Bristol General Hospital, Lecturer on the Practice of Medicine, &c.; Retrospective Address in Surgery, from July 1836, to July 1839, by J. H. James, Esq. Surgeon to the Devon and Exeter Hospital; Observations on the Variolæ Vaccinæ, as they occasionally appear in the Vale of Aylesbury, with an account of some recent experiments in the Vaccination, Retro-Vaccination, and Variolation of Cows, by Robert Ceely, Esq. Surgeon to the Buckinghamshire Infirmary, (illustrated by engravings from original drawings.)

From this enumeration it will be seen that the purely medical articles are limited to the subject of vaccination.

The Report of the Section appointed to enquire into the present state of vaccination has been considered with care, and is reported with ability. It is too voluminous and too circumstantial to admit of analysis on our part, and all that we can do is to select some of the important statements it contains, and put our readers in possession of them. It is gratifying to be enabled to add that they tend eminently to fortify our confidence in vaccination. The Reporters observe:—"The first division of the report embraces those points that have received what we take to be a satisfactory demonstration. The other divisions, all more or less dependent upon it, have been drawn up from authentic documents, and set forth the past and present experience of many of the most respectable professional gentlemen in this country, in a faithful and condensed form; this at least has been our aim. It must, at the same time, be admitted, that the testimony has not always accorded. In arriving at conclusions we have been compelled to weigh and balance evidence; but as the facts which have guided us are fairly stated, our opinions can at once be brought to the test, and of course will avail no more than as they appear to be conformable to the truth. With this admission we desire it to be understood that nothing has been introduced of an hypothetical or speculative nature; nothing has been kept back unfavourable to the cause of vaccination; neither has anything been withheld that was calculated to produce an impression of a different kind." They subjoin their programme:—"We shall now state the order in which we mean to arrange the different heads or sections. The first will treat of the affinities between human small-pox and cow small-pox. The second will contain what we deem essential to render vaccination correct, with some observations on the impediments to that practice. We will then proceed to consider the protecting power of vaccination. Our fourth section will comprise remarks on small-pox after small-pox; which will be followed by a brief examination of the question of re-vaccination. Lastly, we shall enquire into the state of the population generally, both with regard to small-pox and vaccination; the means at present employed for the dissemination of the former, with suggestions for restraining it, and promoting genuine effectual vaccination."

### AFFINITIES BETWEEN SMALL-POX AND COW-POX.

The reporters remark with cordial exultation, that the great problem respecting the nature of the security afforded to man by the communication of the vaccine disease, is solved. And they pay a high compliment to Jenner's sagacity for having insisted on the probable identity of the vaccine and variolous diseases.

The reporters refer to various epizootics which prevailed from time to time, and were of the nature of variola. Horses as well as cows were liable to the affection. "This interesting fact," they say, "illustrates and explains one of the most difficult and perplexing events in the practice of vaccination. It was known that a disease from the horse was sometimes communicated to the cow by men employed in dressing the heels of the one, and afterwards milking the other. This disease was supposed to be what is vulgarly called *the grease,* and was imagined by Dr. Jenner, in the outset of his enquiry, to be the origin of small-pox. This idea he lived to correct; but the prejudices it excited, and the erroneous views to which it gave birth, have unhappily been perpetuated. It is ascertained that the horse is liable to a vesicular disease of a variolous nature as well as the cow; and that lymph taken from the horse and inserted into man, will produce an affection in all respects like that derived from the cow, and equally protective. The error consisted in believing that this affection was *the grease,* and that it required to be transmitted through the cow to give it efficacy. A misapprehension of this kind may well be excused in the infancy of so complicated an investigation. The disease appearing for the most part on the thin skin of the heels of the horse, and the traditions among the farriers in the country, leading to the mistake. We now know that the vesicle may appear on other parts of the animal's body; and that the horse as well as the cow has, in different ages and in different countries, suffered both from the mild and malignant *variolæ.*"

The reporters cite some facts for the purpose of shewing that the variolous disease in the cow may be very aggravated as well as mild, and communicate to man an equally aggravated as well as mild distemper. Had inoculations, they are of opinion, been performed on man with lymph taken from the cattle as they were afflicted in the middle and latter part of the last century in England, we should probably have witnessed the very same results that have recently taken place in Bengal.

A point on which the reporters insist is the simultaneous existence of small-pox among men and the lower animals in this country—a circumstance observed on many occasions, and indeed not in this country only, but abroad. The facts which they adduce, have all but proved, say the reporters, that the vaccine disease is not a preventive of small-pox, but the small-pox itself. In order to complete the demonstration, it is necessary to show that the *human small-pox* can be communicated to the cow in like manner as the disease of the latter has been communicated to man. There are good grounds for believing that many of the variolous pestilences that have at different times laid waste the various regions of the earth have been common both to man and the brutes. After noticing several experiments, apparently successful, but not hitherto considered with the attention due to them, the reporters remark :—

"What many gentlemen in this country failed to accomplish, we are happy to say has been at length achieved by one of the members of our Association, Mr. Ceeley, of Aylesbury. Influenced by some of the facts and reasonings mentioned above, he resolved to attempt to ascertain whether he could, by inoculation, impregnate the cow with human small-pox. Twice he has succeeded in accomplishing this important object after many previous fruitless trials. His experiments were conducted in the presence of five medical men and one veterinary surgeon. He produced five vesicles on the cows, from which source several hundred patients have been vaccinated, who have exhibited all the phenomena of vaccination in the most perfect form and complete degree ; there was no attendant eruption, nor any thing that could lead him to suspect that he had not in this manner propagated the genuine *Variolæ Vaccinæ.* He kindly transmitted portions of this lymph to the President of the Section, who immediately committed it to the care of Mr. Coles and several other gentlemen, in whose hands it produced the most regular vesicles, which in every respect corresponded with those so beautifully delineated by Dr. Jenner, in his first publication. This circumstance forcibly arrested the attention of every one who saw the vesicles, and that too, in several instances, though the source whence the lymph was derived was not known. The correctness of the vesicle formed by it, exhibits a marked contrast to that which we have seen produced by other virus now in use ; and we fear that the local as well as general disturbance occasioned by the latter, so far from being a source of protection, will be found to be the reverse." 26.

On the 1st of February 1839, he inoculated with small-pox matter (*variola discreta*) of the seventh or eighth day, three young heifers ; a fourth was at the same time vaccinated. The reporters limit their account of what happened to the first.

"Mr. Ceeley made seven punctures and introduced fourteen points near the left *labium pudendi,* and on the same day inserted two setons with matter from the same subject. On the ninth day after this process, he vaccinated the same animal on the right *labium pudendi,* with fifth, sixth, and seventh day's lymph from a child, in seven punctures with fourteen points ; and below the pudendum in four punctures with eight points. On the tenth day after the insertion of the variolous matter, one of the punctures near the posterior margin of the left *labium pudendi* had assumed the form of the natural vaccine vesicle. By gently removing the central irregular crust, and carefully puncturing the cuticle, he was able, in the course of an hour, to charge thirty-eight points with lymph, and on the same and subsequent days to use part of it on children and adults. On the thirteenth day the small-pox vesicle was more inflamed and florid ; this was the fifth day after the insertion of vaccine lymph, at which time all the eleven punctures were converted into effectual vesicles ; from these he took fine clear lymph, and used it on children and adults. Both the variolous and vaccine vesicles subsequently ran nearly a parallel course ; so that on the twenty-sixth day of the former, and the seventeenth day of the latter, the scars of both appeared perfectly similar.

To obviate objections which might arise from the insertion of the vaccine lymph on the ninth day after the inoculation with the variolous matter, Mr. Ceeley re-inoculated a sturk on the 15th of February with small-pox matter, of the seventh or eighth day, on the *labium pudendi*. He made eight punctures, which were deluged with the variolous fluid from capillary tubes. On the fifth day the four upper punctures were enlarged and elevated; the other four were less so. On the sixth day all presented the appearance of the vaccine vesicle. From one of them he took lymph with difficulty, and scantily charged thirty-nine points. On the eighth day he again took lymph from the vesicle opened on the sixth. On the ninth day the vesicles were enlarging, and he again opened carefully the first vesicle and charged twenty points. On the tenth day the four lower vesicles were increasing, and from them he charged twenty-seven points. After this time the brown crusts appeared, and the disease gradually declined. This animal was subsequently inoculated both with variolous and vaccine matter, but no result followed.

The practice of inoculating the cow with vaccine virus taken from man was very early attempted. It is not to be overlooked that the difficulty of accomplishing this, is almost as great as that of inoculating the animal with small-pox. It succeeded however in the hands of Dr. Waterhouse, who, in 1801, impregnated one of his cows, and obtained from her a ' crop of matter on the ninth day, which produced the disease in the human subject to perfection.' Mr. Fox, of Cerne Abbas, who has paid great attention to this subject, and seen the disease, as well among the cattle as on the hands of the milkers, has also successfully vaccinated the cow.

The same experiments have been performed at Passy, in the neighbourhood of Paris; the lymph found there in 1836, among the cows, has been recently again passed through the animal, and this is called retro-inoculation. Mr. Ceeley, too, has often been enabled to communicate the vaccine disease from man to the cow. He has observed that good human lymph, when transmitted in this manner, loses some portion of its activity; it rises late, and produces smaller vesicles, but ultimately, *after successive innoculations on man*, it resumes its activity." 28.

The Reporters rightly designate this a triumphant conclusion of an investigation of more than fifty years' duration, and the best monument to the sagacity of Jenner. They deduce from what has been done the following aphorisms, to which they entreat the attention not only of the profession, but of the public.

First, that cattle in many ages and different countries, have been afflicted with small-pox.

Secondly, that this disease among the inferior animals has simultaneously existed with the small-pox in man, and pursued its victims through every quarter of the globe; and that it exists at this time in Asia in a fatal and pestilential form.

Thirdly, that it appeared among the cattle in England in the year 1745, and again in 1770, and continued its ravages up to the year 1780; and that the local remains of this epizootic occasionally still shew themselves with considerable severity.

Fourthly, that the casual transmission of this disease to the milkers in the dairies of Gloucestershire, and their subsequent immunity from human small-pox, first led Dr. Jenner to the investigation of this singular affection, and ultimately to establish it as a substitute for the more pestilential and fatal form of the disease.

Fifthly, that when the disease appears among the inferior animals in a malignant form, it produces, by inoculation, a disease of similar severity in man.

Sixthly, that as man has received this affection from the cow, so likewise has the cow received it from man.

Seventhly, that the direct inoculation of the cow with human small-pox, has produced a mild and mitigated disease; and that such disease, reproduced by inoculation on man, accords entirely in its character, in its progress, and in its protecting influence, with the *variolæ vaccinæ*, as described by Dr. Jenner; thus irresistibly proving his fundamental proposition, that cow-pox and small-pox are not *bonâ fide* dissimilar, but identical, and that the vaccine disease is not the preventive of small-pox, but the small-pox itself,—the virulent and contagious disease being a malignant variety.

### Correct Vaccination and Impediments thereto.

The Reporters observe on the discrepancy that obtains in the statements and ideas of medical men upon this head.

1. The only perfect test is the insertion of variolous lymph. This, however, is obviously objectionable.

2. The regular progress of the vaccine vesicle. To determine this, the surgeon should note it at the proper periods. The genuine disease can only be produced by pure lymph from a regular source. The time for taking this lymph, according to Doctor Jenner, is between the fifth and eighth days, and before the formation of the areola. Others have recommended the use of lymph taken at a much later period; but this they believe to be a very questionable practice.

3. Jenner proposed that some fresh vaccine lymph should be inserted into the patient a few days after the first vaccination. This practice was founded on the observation that the second vaccination proceeds with accelerated speed, provided the first has taken effect. It is a very simple and beautiful illustration of the constitutional effects of vaccination, and deserves to be encouraged. An experienced eye will for the most part be able to detect any deviation from the true vesicle.

4. The character of the lymph employed. It never ought to be taken from a vesicle which deviates in the least degree from the perfect standard, nor from a patient labouring under any cutaneous disease.

5. A point which ought ever to be insisted upon, is the leaving one or more vesicles to run their course without being in any way disturbed.

6. The appearance of the cicatrix. The Reporters think that this has been too much trusted. They are inclined to believe that though the presence of a perfect cicatrix is not a sure sign of protection, its absence must be held to speak strongly against the existence of vaccine influence. Yet the observations of Mr. Dodd would seem not to bear out this impression. Of fifty-seven cases that had been exposed to the contagion of small-pox and escaped, in six only was the cicatrix perfect; in fourteen it was slightly defective; in thirty it was very imperfect; and in seven it was totally wanting.

Out of seventy-seven cases of small-pox after vaccination, one bore a perfect mark, fourteen had the cicatrix slightly defective, forty-seven were imperfect, and fifteen had none at all. Thus, to sum up the whole, out of one hundred and thirty-four cases of vaccinated persons who had been exposed to small-pox, the cicatrices of seven were perfect, and one of these failed; twenty-eight slightly defective, of which fourteen failed; seventy-seven very imperfect, forty-seven of which failed; twenty-two had no marks at all, and of these fifteen had small-pox, while seven altogether escaped.

7. The Reporters think, that vaccine lymph, though passed through a great number of subjects, and used for a great number of years, does not necessarily become deteriorated. This, however, can only be said when unceasing attention is paid to every successive transmission; for if a deviation commences, it may be perpetuated, and afford a gradually decreasing protection. There is no doubt that lymph of this kind has been often used.

8. The influence of cutaneous diseases on the vaccine vesicle has been, the Reporters think, insufficiently attended to. Dr. Jenner pointed out that the affection was very much modified in its progress by the scaly tetter, and those affections described by Dr. Willan under the term *psoriasis*, as well as those vesicular eruptions commonly called herpetic. He observed that vaccination performed on a skin occupied by any of these diseases, " produces every gradation from that slight deviation from perfection, which is quite immaterial, up to that point which affords no security at all." The Reporters recall these observations with much earnestness.

9. " Other constitutional peculiarities stand in the way both of human small-pox and cow small-pox. Some resist these affections at one period of their lives and not at another; and there are examples of the very opposite condition, which show that the individual will receive either infection as often as it is presented to him. These peculiarities frequently run in families. We know several children of the same parent, who have had modified small-pox after cow-pox; and not many months ago three brothers had small-pox after small-pox, one of these cases proving fatal. On this subject we have illustrations from Mr. Dodd, who tells us that six brothers and sisters in one family having been vaccinated when children, had the small-pox a few years afterwards. In another instance, two sisters vaccinated in infancy, were subsequently inoculated and had small-pox slightly, they both caught the small-pox again in 1837, and one of them had it very severely. Their father caught small-pox; their mother too, who was inoculated when young, had it again in the same year; their maternal grandfather, beholding from a window at night, the funeral of a friend who had died of small-pox, sickened of that disease and died. These are a few of the affinities and concordances between human small-pox and cow small-pox; and we doubt not that every subsequent observation will establish the analogies. In confirmation we farther remark, that the great object of inoculation with human small-pox, was to produce an affection as much like that of cow small-pox as possible, and by great care in selecting the virus to be employed, this was sometimes accomplished in a very remarkable degree. On the other hand, it is known that the disease, when casually caught from the horse or cow, is often a severe one,—as severe, it was said by an experienced observer, as for the most part was inoculated small-pox. We ourselves have seen it, when caught from the horse, exhibiting great intensity, the hands and arms being covered with the eruption." 39.

### Protecting Power of Vaccination.

The Reporters present many statements from different sources on this head—a few discouraging, the majority the reverse. The question must still be regarded as unsettled, though we do confess that, while we fear the protecting power of vaccination has been estimated by the enthusiastic Jennerians too high, the general results are greatly in its favour. The Reporters, indeed, look on all as *couleur de rose.*

"From an impartial review," say they, "of the whole of the evidence submitted to us, we are called upon to declare that, with one exception, we do not find any thing to authorise statements respecting periodical failure. That the protecting power of cow small-pox may disappear, must be conceded; but this is no more than occurs with those who have had human small-pox, for they are alike subject to a second attack of the same disease. After the demonstrations already given, we feel ourselves called upon to stand firmly upon this ground. Opinions, which formerly admitted only of analogical illustration, have now received direct and positive confirmation, and we, therefore, hold it to be proved beyond all doubt, that the same general laws which govern *human small-pox,* apply, '*mutatis mutandis,*' to *cow small-pox.* We have a great weight of testimony, all entitling us to assert that the cow small-pox, duly and efficiently communicated to man, does not lose its influence by time. From personal observation, we are entitled to speak with considerable confidence on this point; and many of our correspondents who have been longest acquainted with the practice of vaccination, tell us that they have met with nothing leading them to believe that such a law exists with respect to the disease." 60.

The Reporters feel themselves constrained to ask, when they hear of a great number of cases of small-pox after vaccination:—

1. Has the lymph been pure and perfect?
2. Has the development of the affection been regular and complete?
3. Has the state of the recipient, both with regard to the condition of the skin and other constitutional peculiarities, been such as to present no impediment to the regular course of the affection?

They protest that,—All cases of reputed vaccination, unless they have passed under the review of a competent judge, who has witnessed the different stages of the affection, should be considered as no vaccinations at all. And, finally, they affirm that:—cow small-pox and human small-pox, we repeat it, are alike in their general properties; and if the latter, once taken, for the most part prevents subsequent attacks, so, in like manner, does the former.

### Small-pox after Small-pox.

Of the not unfrequent occurrence of this no doubt can now be entertained. The Reporters accumulate instances of it. But it is not necessary for us to follow them. Suffice it to quote the conclusion they arrive at:—If we except the fatal cases of small-pox after vaccination which are reported to have occurred in the Small-Pox Hospital in London, the deaths from small-pox after small-pox in the country correspond very nearly with the deaths from small-pox after reputed vaccination. This coincidence is more deserving of notice, when it is considered that small-pox of late years has been very much controlled, and infinitely less diffused than it was before the introduction of vaccination.

### RE-VACCINATION.

The Reporters think it important to inquire whether a successful re-vaccination proves that the individual who is the subject of it, is thereby shown to have lost his vaccine protection, and, consequently, to have been liable to small-pox infection. That idea appears to be unfounded. The Reporters "know individuals vaccinated many years ago, who, last winter, as well as on many former occasions, were exposed to small-pox contagion, and resisted it. A very short time after such exposure, they were re-vaccinated, and a complete vesicle appeared. Again the converse is shown by a circumstance which has recently come to our knowledge. A clergyman who was vaccinated in his infancy, was, about ten months ago, re-vaccinated, and completely resisted the infection. Two months afterwards he was exposed to the contagion of small-pox, caught the disease, and had it somewhat severely. We know likewise of cases in which small-pox followed re-vaccination; one took place a few weeks after that process; another is mentioned by Mr. Bailey, of Thetford. A pupil of his was vaccinated when an infant; he attended many severe cases of small-pox, and fearful of the infection he re-vaccinated himself, and went through the disease well. Last winter, while at Guy's Hospital, he took the small-pox, had it smartly, and was obliged to leave town for the country, to recover his health.

We know another case that deserves to be noticed. A lady was vaccinated in 1806. She was re-vaccinated in 1833, and resisted the infection. In 1839 she was exposed to the influence of small-pox in a house where a child died of that disease. On this occasion she was seized with a violent incursive fever, which was succeeded by a slight variolous eruption, but it died away in a few days. It may be mentioned, at the same time, that her sister, who was vaccinated in 1809, had also a slight attack of small-pox, having had about thirty pustules."

Sometimes, after a person has had small-pox, a local effect may be produced by every insertion of the variolous matter, and the Reporters believe that, in like manner a vaccine vesicle may be obtained. They insist upon the fact, that re-vaccination succeeds or otherwise on persons who have had small-pox or cow-pox, almost exactly in the same ratio; thus establishing another most remarkable analogy.

The proportion of one hundred cases of each description is as follows:—

| | | | |
|---|---|---|---|
| Vaccinated after small-pox, | with success | .. .. .. | 32 |
| Ditto ditto | modified | .. .. .. | 26 |
| Ditto ditto | without effect | .. .. .. | 42 |
| | | | 100 |

| | | | |
|---|---|---|---|
| Re-vaccinated, | with success | .. .. .. .. | 34 |
| Ditto | modified | .. .. .. .. .. | 25 |
| Ditto | without effect | .. .. .. .. | 41 |
| | | | 100 |

They contend that what occurs in this country affords no evidence of the decay of the prophylactic powers of vaccination, although the statements they have obtained upon the subject are conflicting. They are of opinion that re-vaccination can only be required where doubts are entertained of the correctness of the first vaccination. And they add:—

"Systematic re-vaccinations appear to us uncalled for, and liable to several objections, which we will now briefly state. In the first place, the practice implies that the virtues of cow small-pox are less permanent than we believe them to be; and now that this point has been freed from all ambiguity, we are not inclined to do any thing to shake the confidence which must ultimately spring from right views of this subject. In the next place, it is probable, if re-vaccination be looked upon as essential, that less attention may be paid to the first vaccination than it demands, persons believing that all imperfections may be rectified by the subsequent operation. Now, as we are firmly convinced that incomplete vaccination has been the cause of a large proportion of failures, we cannot help dreading that defects of this kind, which it is so needful to remedy, might become more frequent than they are at present."

State of the Population with regard to Small-pox and Vaccination: suggestions for restraining the former and promoting the latter.

Formerly, in Great Britain and Ireland, between forty and fifty thousand were supposed to perish annually from small-pox; and if we take into account the vast increase of the numbers of the people, probably eighty thousand or more would now fall a prey to it, but for vaccination. From the Registrar-general's First Annual Report it appears, that in the half-year ending on the 31st of December, 1837, one hundred and forty-one thousand six hundred and seven deaths were registered in England and Wales. Of these, five thousand eight hundred and eleven were occasioned by small-pox. The disease was epidemic, particularly in Liverpool, Bath, and Exeter. Of one thousand and fifty-six deaths registered in Bath, Liverpool, Exeter, parts of Shropshire, Worcestershire, and the Metropolis, eight hundred and eighty-seven occurred under the age of four years; ninety-nine between five and nine; fifteen between ten and fourteen; eighteen between fifteen and nineteen; twenty-nine between twenty and twenty-nine; five between thirty and thirty-nine; two between forty and forty-nine; one between fifty and fifty-nine. This specification, it is added, may be considered as an approximation to the ages of the five thousand eight hundred and eleven; and it is inferred that the majority of this number had never been vaccinated, as so large a proportion of deaths occurred among the very young; and it is known that the poorer classes, if they do not neglect vaccination altogether, often defer it for years. The Reporters' returns bear out this view of the subject. It appears, however, that there are only four diseases more fatal than the small-pox. Nearly six thousand perished in England and Wales in six months; and if the same rate of mortality existed in Ireland and Scotland, at least ten thousand victims might be counted within that period.

It is creditable to the profession that, with only one or two exceptions, it refuses to inoculate small-pox. It would be disgraceful in the highest degree could such a stigma attach to many, or to any of us.

The small-pox inoculators are obscure, ignorant and mercenary persons, who pander to the fears and prejudices from which they draw their disgraceful earnings. Those prejudices are far from weak. At Collumpton, according to Mr. Maunder, small-pox inoculation is extensively practised, and it is difficult to get the people to submit to vaccination, they generally preferring (to use their own expression,) "*the real thing*." Mr. Fox, of Cerne Abbas, tells us that, in a multitude of instances, the poor prefer paying from sixpence to two shillings per head for small-pox inoculation. His neighbourhood is visited periodically by small-pox inoculators. Last year they had a dissenting preacher, a blacksmith, a miller, and an old woman, all taking up this murderous trade; and can it be surprising that there were upwards of three hundred cases of small-pox? Yet under circumstances so unfavourable, he adds, "I have not seen many cases of small-pox after vaccination, and only four within the last year."

The Reporters proceed to contrast the regulations with respect to vaccination on the Continent with our own—not much to the advantage of the latter. And then they propose certain measures to the Legislature, with the view of excluding vaccination and repressing small-pox.

The Reporters make several propositions based on the facts to which we have alluded with more or less circumstantiality.

1. This relates to the practice of small-pox inoculation. "It is well known," they say, "that the exposure of a person labouring under a contagious disease is at present an offence at common law, and punishable by fine and imprisonment. Several convictions have of late years been obtained in consequence of the violation of this law by persons affected with small-pox; but the actual practice of small-pox inoculation does not incur any punishment, though it certainly ought to do so. Now, it has been shown that the diffusion of small-pox epidemically in many districts, may be distinctly traced to individuals not connected with our profession, who have taken up the small-pox lancet, reckless of the consequences, and have thus disseminated the contagion to a most alarming extent. Such persons, we think, ought to be restrained. We therefore propose that the legislature should be petitioned again to declare that individuals labouring under small-pox shall not, under any circumstances, be exposed in such manner as to lead to the dissemination of the disease; and, moreover, that none but a regularly educated medical man be permitted to inoculate for small-pox, and that he likewise shall be liable to all the penalties which may attach to the infringement of the statute touching the incautious exposure of persons labouring under the disease."

2. They would recommend that epizootic diseases, and the history and character of the *variolæ vaccinæ*, should be made subjects of particular instruction and investigation in every school of medicine throughout the kingdom; and that candidates for degrees in medicine and surgery should be specially examined in this branch of professional knowledge, and that they should likewise have opportunities of practically witnessing and understanding all the instructions they may receive from their teachers.

3. They would propose that duly qualified vaccinators should be appointed for every district in the kingdom, whose main duty should be, at certain seasons, to offer gratuitous vaccination to the poor of every hamlet and village or parish within their respective bounds. The stations and days and hours for vaccination might be fixed, and also the times for inspecting the progress of the affection. Registers, accurately constructed, would show every circumstance connected with perfect or imperfect vaccination, which could always be referred to on any future occasion when small-pox made its appearance in the neighbourhood.

Another duty, they observe, of considerable moment might easily be attached to the offices of these public vaccinators. Living in rural districts, and instructed in the diseases of the inferior animals, they might take advantage of the appearance of the *variolæ*, either among cows or horses, and procure fresh supplies of lymph when such should be required; or they might perform such experiments as have been successfully conducted by Mr. Ceeley for the renewal of the disease.

We cannot forbear adding one passage. It bears upon one of those evils which have been grafted on the New Poor Law, in consequence of the mistaken and mischievous view entertained by its promoters on the subject of medical relief. It is to be hoped that cruel practices, such as have been seen, upon the poor will soon have ceased.

"We have very strong statements from many of our correspondents respecting the unprotected state of the poor, especially since the New Poor Law came into operation. The Association is too well acquainted with the bearing of that statute upon the medical relief provided for the pauper, to require us to dwell upon any other part of the subject than that which we have immediately in hand. Formerly a moderate and just allowance was made to the parish surgeon for vaccination. In many cases that allowance is either withheld entirely, or so much diminished in value as to cause a very unfortunate neglect of this salutary practice. One gentleman in Cambridgeshire tells us that he had practised thirty-eight years. He seldom passed two years without vaccinating the whole parish: the consequence was, that small-pox was scarcely known, and the mortality as trifling, perhaps, as in any other place of its size in England. The Guardians of the Poor now only allow an insufficient remuneration, and he therefore infers that vaccination will be neglected, and that should small-pox appear in such districts, he significantly adds, the cost of coffins alone will be a heavier charge upon the parishes than would have been incurred by paying a proper remuneration to the surgeon for averting this calamity by vaccine inoculation. Dr. Jenner himself found this very argument (viz. the cost of coffins,) influential in driving certain parishes in the neighbourhood of Cheltenham to seek for vaccination.

The Reporters conclude by expressing their satisfaction at the general tenor of the data they have collected in reference to vaccination—data upon the whole extremely favourable to it—and by an eulogium scarcely, perhaps, less just than warm, on the foresight and sagacity of Jenner.

OBSERVATIONS ON THE VARIOLÆ VACCINÆ, AS THEY OCCASIONALLY APPEAR IN THE VALE OF AYLESBURY, WITH AN ACCOUNT OF SOME RECENT EXPERIMENTS IN THE VACCINATION, RETRO-VACCINATION, AND VARIOLATION OF COWS. By *Robert Ceely*, Esq. Surgeon to the Buckinghamshire Infirmary.

Mr. Ceely's paper has been already referred to. It is highly creditable both to his zeal and his ability, and is calculated to add great clearness to our notions of the vaccine disease. We shall allude to such parts of it as seem of most direct importance.

After describing the topography of the Vale of Aylesbury, Mr. Ceely runs over the endemics, epidemics, epizootics, and enzootics that obtain in it. He then describes the variolæ vaccinæ as they appear there, both in their regular and their irregular form. He then passes to the *casual cow-pox* in man.

Speaking of this, Mr. C. observes that—although the casual cow-pox in man is mostly found on those who have not previously gone through variola or the vaccine, it is by no means rare to meet with it on persons who have passed through the latter, and a few who have had the former disease. It is no novelty to see individuals who have taken the casual disease more than once, at various intervals, but not severely; and now we often see cases after vaccination, at periods of from two to fifteen years, of different degrees of severity, not always, but often proportioned to the time elapsed, many declaring their symptoms to be more distressing than those which they remembered of the previous vaccination. On the other hand, we now and then meet with persons who, without any protection, have used every endeavour to acquire the disease by milking, but have failed amidst their more fortunate fellow-labourers.

Mr. Ceely describes the disease as it appears, and the anomalous or irregular forms that it presents. He concludes that milkers are liable to small-pox—first, from having been the subjects merely of the spurious diseases; secondly, from imperfect vaccination, at a late period of the disease, with *deteriorated* and purulent virus, or even with *perfect* lymph, of which they had not at the time been sufficiently susceptible.

Mr. Ceely makes some good and valuable observations on the *primary lymph.*

To procure this liquid, and fit for use, is no easy matter. Mr. Ceely remarks that—acuminated vesicles, containing a thin, serous, and straw-coloured fluid, and of course of little value, more commonly vesicles broken and nearly destroyed before that period has arrived, are often all that remains. Often, too, there are no crusts present or in prospect that can be depended upon. Unless the disease arises in a large dairy, and a consequent succession of cases occurs, it will seldom happen that vesicles are found capable of yielding that which is sought for. More frequently than would be suspected, the practitioner will have the mortification to find on his arrival, that, from the lateness of his information, a whole dairy of cows has passed through the disease, and he can scarcely find even a useful crust. The hands which have damaged the vesicles have also prematurely removed the crusts. Still a careful inspection of all the subjects should never be omitted; *primary* crusts should be sought for on the lower part of the udder and around the base of the teats. During this search it is not improbable that small vesicles of later formation may be found yielding efficient lymph.

Mr. Ceely gives precise directions for obtaining the best lymph.

"It is to be got from perfect vesicles before the period of acumination; after this period it is less to be depended on, particularly if very abundant, thin, or discoloured. Taken immediately before acumination, it is quite as active as that of an earlier date. In the earlier period, from the fifth to the ninth days, it is often scarcely possible to obtain it in any quantity except the skin of the part be very thin and lax. Lymph from a vesicle which has not acuminated, even if turbid, should not be rejected; it will often succeed when limpid lymph from an acuminated vesicle will fail. Acuminated vesicles which have been broken are rarely to be relied on; and vesicles which have been broken at an earlier period are not much better, and, on account of their liability to afford more than the specific secretion, may prove highly objectionable to some individuals. Entire unacuminated vesicles, or vesicles with central crusts, should be sought for where they are least exposed to injury, viz. on the lower and naked parts of the udder, and the adjoining bases of the teats; but as there is some difficulty in obtaining lymph in sufficient quantity from these sources, the greatest care and circumspection is necessary. It is impossible to exercise too much delicacy in the proceeding. It is scantily secreted, as before stated; the exudation of blood will obscure, and the sensitiveness of the animal under a rough manipulation sometimes altogether prevent, the process. The puncture should be made with a sharp lancet as near the centre of the vesicle as possible, and the epidermis be gently raised to a moderate extent around the discoloured or most depressed part. Slight pressure, either with the blade of the instrument or between the thumb and finger, if done with tenderness, will enable the operator, after some time, to charge a few points with the slowly exuding lymph. Patience and a treble charging of the points are always to be recommended. From what has been stated in another place, it will be understood that the puncture at the elevated and indurated margin of the vesicle will be perfectly useless, as it yields only blood; it may be pernicious by irritating the animal.

Vesicles with central crusts will be found perhaps more convenient, and nearly as productive as those just mentioned, particulary if the crust be small, and the margin of the vesicle tender, hot, and tumid. The crust may be wholly removed or pressed on one side, the epidermis underneath may then be carefully raised if the lymph does not soon exude, and a perfectly limpid fluid, in small quantity, can thus be procured.*

When we are, as too often happens, deprived of the above advantages, we may yet succeed in procuring useful substitutes for liquid lymph in—first, amorphous masses of concrete lymph; secondly, central irregular crusts: thirdly, vesicular crusts or desiccated vesicles. The first are often found upon or in close proximity to broken vesicles; and are either colourless, like crystals of white sugar candy, or of a light amber colour, resembling fragments of barley-sugar, according to the period of their escape, and the length of time they have been exposed to the air. With a few drops of cold water they may be reduced again to the liquid state, and employed with a probability of success. The central rough and irregular crusts, often more or less conical, occasionally contain an admixture of blood or some discolouring secretion, or other adventitious

* "Small superficial vesicles are often more yielding than contiguous larger vesicles more deeply seated."

superficial coating: the more transparent and the nearer a dark brown the better. Desiccated vesicles should be carefully abstracted by the milker before they are casually removed or spontaneously fall, and those only of primary formation, the model of a vesicle, dark-brown and somewhat translucent, should be retained.

Secondary and tertiary crusts are of scarcely any value: these are more or less thin, ill-defined, or irregular in form, of a dirty yellow or yellowish-brown, more transparent, though not unfrequently more dusky and opaque, containing a large admixture of concrete blood and pus." 341.

Mr. Ceely remarks, in conclusion, that good crusts, treated in the manner first suggested by Mr. Bryce for human vaccine crusts, may as frequently be depended on, in suitable subjects, as liquid lymph, and more frequently than much of that which is commonly met with. He owns that, after all, we must not be too fastidious as collectors, success as well as failure not unfrequently occurring when we do not expect it.

*Vaccination of Man with Primary Lymph.*

Mr. Ceely points out the circumstances which preclude the frequent practice of vaccination with primary lymph. But were it far more practicable than it is, there are formidable obstacles to its *success*—consisting, first, in the difficulty with which animals of one class receive a morbid poison generated in animals of another class; and secondly, in deficiency or imperfection in the mode of communication. Mr. Ceely presents the following summary of his experience:—

"1. More than half my attempts to vaccinate with primary lymph, taken from vesicles at a proper stage, and possessing all the characteristics of perfection, have entirely failed. The same individuals have immediately afterwards been successfully vaccinated with dry or liquid lymph which had been long current in man.

2. A small number, vaccinated from the same primary sources, afforded results in various degrees of imperfection. In general, the seventh or eighth day has elapsed before any indications of infection have appeared: a dark red pimple has slowly advanced, and gradually assumed a tubercular or tuberculo-vesicular form, being either perfectly lymphless, or a dark red tubercle on the thirteenth or fourteenth day with a scanty supply of lymph on its acuminated summit, with a moderate areola, slowly subsiding, attended by very trifling or scarcely appreciable constitutional symptoms. Nearly all these subjects have been successfully re-vaccinated with ordinary lymph, from periods of nine to eleven months in different degrees of modification, but always with a nearer approach to perfection.

3. A still smaller number, vaccinated from the same primary stocks, have furnished vesicles in the highest degree of beauty and perfection. But even in many of these there has been more or less delay in the full development of the vesicles; and in nearly all, the number of the vesicles has seldom equalled one-half of the punctures.

4. Precisely similar phenomena of entire failure, imperfect, or complete vaccination, with all their attendant circumstances, have followed the use of lymph from perfect casual vesicles on the hands of milkers; and the like results have frequently attended the early removes of lymph from the most perfect primary vaccinations.

5. The whole of these phenomena—abortive punctures, tubercles, tubercular vesicles, imperfectly developed, and, perfectly normal vesicles—may be frequently seen co-existent on the same individuals, either from primary lymph, casual lymph, or the early removes of both from any given vesicle.

6. Although, with the few exceptions previously stated, the copious, repeated, and long-continued casual application of cow-pock lymph to a highly sensitive, vascular, mobile, and extended surface, with or without abrasions, chaps, or fissures of the cuticle during the process of milking, proves a more perfect and successful mode of communicating the disease, yet it is abundantly obvious that even this superior mode is in general more prompt and certain in infecting the cow than the milker." 345.

Mr. Ceely gives a very explicit account of the phenomena observed after vaccination with primary lymph. We must refer the curious to his report. But we may observe that the vesicles admit of remarkable improvement by transmission of the lymph through a series of well-selected subjects. By this process, also, in a very short time, most of the defects and some of the evils connected with the use of primary lymph may be dissipated, and the lymph rendered milder and more suited to general purposes.

*Vaccination of the Cow with Primary Lymph.*

Mr. Ceely was desirous of ascertaining the susceptibility of the sturk (or young heifer) to the disease artificially communicated. He commenced therefore, by vaccinating some of these animals, about ten months old, in the inside of the ear, on the teats, and in the soft and vascular structure on and near the vulva, with lymph taken from milch cows in the winter of 1838-9, while the disease was prevalent in many of our dairies. Punctures were made in the ordinary way with lancets well charged and with points thoroughly imbued with good lymph; two or three of the latter were broken off short, and allowed to remain in each wound. He found much less difficulty in succeeding than he expected, or than is experienced in vaccinating those animals with lymph taken from man. The lymph used was from the same source and of the same date as some which had been applied to children and adults. On these last, however, it either failed altogether, produced more or less imperfect vesicles, or perfect vesicles with the areola not fully developed till the twelfth, thirteenth, or fourteenth, and fifteenth or sixteenth days.

*Vaccination of the Cow with Humanized Lymph, or Retro-Vaccination.*

"My experiments in retro-vaccination were almost entirely confined to the sturk under twelve months old; and the parts selected for the operation were those in close proximity to the vulva. These animals were easily procured, while milch cows in any number, at least here, are difficult to obtain for such a purpose. The parts chosen are most easy of access, and in every respect, I think, most desirable. The operation was performed either with a lancet or a sharp straight bistoury. The lymph was either dry, or liquid in capillary tubes. No other precautions were taken than excluding the animals from wet and cold immediately after the operation for a few hours or perhaps a night. Those of a light colour and with thin skins were generally preferred, but often without avail, scarcely one half of the operations succeeding. Tubercles, nearly or completely lymphless, were often produced; but sometimes every puncture succeeded. In the majority of instances the vesicles ran the normal course, declining on the eleventh day. Four kinds of lymph were at different periods employed, each having been current in man for a longer or shorter period." 355.

Mr. Ceely details four experiments, and comments seriatim on the results. After a good many observations on them, he remarks :—

"The above experiments, however, will serve to show the greater difficulty of vaccinating the cow with humanized than with primary lymph, and that, when successful, a much milder disease is the result. Take an abundance of humanized lymph from one of the finest and most productive vesicles ever seen, and if you succeed in retro-vaccinating the cow, you may perhaps be able to charge scantily only a very few points from a vesicle which excites but trifling topical inconvenience. Vaccine lymph, it is obvious, therefore, in passing from the cow to man, undergoes a change, which renders it less acceptable and less energetic, on being returned to many individuals of the class producing it; some refuse it altogether. But this reluctance or absolute refusal, this difficult imbibition or total insusceptibility, does not exist to the same extent as observed in the converse of this experiment, viz. in passing primary lymph from the cow to man by the same means. Here we have considerable difficulty; but when we succeed, a severer disease is induced. These experiments clearly show that the *age* of humanized lymph does not seem to influence its reception by the cow. Provided that the lymph be of ordinary activity, and possess the normal qualities, it is as likely to succeed in its operation on the animal whether it may have been current in man a few weeks or many years, and will excite equally perfect and productive vesicles. The effects of retro-vaccine lymph on man, as compared with primary and current lymph, are worthy of notice. Lymph which had passed from arm to arm with the greatest promptness and facility, and produced the finest effects, in its first removes from the costive vesicle of the retro-vaccinated cow, is not so readily absorbed by many individuals. It rarely fails: but papulation is retarded, and though the vesicle may attain maturity at the normal average period, the completion of that stage is frequently postponed. The vesicles are often smaller, and the disease really not so well developed, as by the stock from which it was derived. But these changes do not appear after the second or third remove. The lymph is restored to its former qualities, and produces its former effects. Some of the retro-vaccine lymph of the second experiment, transmitted to the Small-pox and Vaccination Hospital, from whence it was originally derived, was passed through a long series of subjects, under the inspection of Dr. Gregory and Mr. Marson, who, though better able to make the comparison, were unable to detect any sensible difference in its local or constitutional effects after the second remove. On the third remove it became 'very fine,' as it was before its transit through the cow." 368.

Mr. Ceely considers the question, whether the practice of retro-vaccination is of any real value as a means of renovating humanized vaccine. He confesses himself unable to discover any advantage belonging to it.

Mr. Ceely, too, examines the question—Is *any* vaccine lymph, in passing through many individuals with all due care and selection, susceptible, in process of time, of actual degeneration or essential diminution of intensity? He points out the circumstance that in India. at Silhet, a speedy degeneration was observed—that M. Bousquet, in France, and Dr. Gregory in London, have also observed a decadence of power in old lymph, and he contrasts with these some opposing testimonies. He winds up by saying—*that* his own repeated applications to the cow have been chiefly for the purpose of experiment, for the satisfaction of patients, or the accommodation of friends, not from any belief in its superior protective efficacy over active humanized lymph. But when lymph is found uniformly deficient in infecting property, vesicles abnormally rapid in their course, at their greatest development on the seventh day, yellowish in appearance on the eighth with turbid lymph, central desiccation on the ninth, and a miserably small crust falling on the fifteenth or eighteenth day, common prudence dictates its discontinuance, and urges the adoption of a new supply, although constitutional symptoms may not be absent, for *weak* lymph may not be better than *late* lymph. There surely, in so delicate and important an affair, must be more satisfaction in using that which will affect promptly and certainly, produce a vesicle in its greatest beauty on the tenth or eleventh day, which will then and even later contain a limpid and often an effective lymph, and desiccate not completely till the fourteenth or fifteenth day. Such we know was the vaccine of 1800.

Mr. Ceely makes a few remarks on the *affinity of cow-pox and small-pox*. The only facts he has been able to collect are these;—" The cow-pox has occurred—First, during epidemic small-pox, but when no cases of variola have been known in the neighbourhood: secondly, when variola has been prevalent in neighbouring towns: thirdly, when variola has been in contiguous villages: fourthly, when in the same village (perhaps a solitary case) in which the affected farm was situate: but, fifthly, I have never succeeded in tracing positive or probable intercourse of convalescents from small-pox, their friends or attendants, with a dairy or its occupants, except in one instance, in the autumn of 1838, during which time, and the early part of the following year, cow-pox was prevalent, and in this case it can scarcely be supposed to have had any influence; the milker had carried a child some miles labouring under modified small-pox;" and lastly, it is often seen in a single farm, but not unfrequently, in several at the same period.

### *Variolation of the Cow.*

Mr. Ceely failed with his first experiments, but, in the early part of 1839, he was more successful. He inoculated three sturks (young heifers) with small-pox virus, (variola discreta), seventh or eighth day, on large points—the teeth of a comb—charged twelve hours previously. These experiments have been already alluded to in the Report of the Vaccination Section, and, we shall merely quote Mr. Ceely's summary of the facts of the second experiment. The first time he inoculated this sturk he failed. But re-inoculation was successful. " It was performed in *part* in a situation where the skin seemed thin and bled freely on puncture. On the fifth day the purplish or livid pimples, so much like the natural or casual vaccine on the thin skin of the teats, at this stage announced the success of the operation *on* the lip of the vulva, and I certainly thought all the other punctures had succeeded on the thicker skin *near* the vulva; the want of colour in these elevations was attributed to the texture of the skin there. On the sixth day every thing advancing, but the larger and colourless elevations seemed without lymph; the four glistening vesicles had a slight central crust, clearly announcing their character. Lymph was procured from one, with great difficulty, perfectly pellucid and adhesive. The seventh day the tubercular character of the four upper elevations quite manifest; they were *subsiding without a central crust;* the four vesicles had increased in breadth, and were less elevated. On the eighth day lymph was again taken from a vesicle which yielded it more readily than others; and, anxious not to interrupt their development, they were not again touched. All the vesicles were of a glassy resplendency; the tubercles were evidently passive. On the ninth day more lymph, perfectly pellucid, very scanty, was taken, the vesicles clearly advancing. On the tenth day, the day of maximum development of the vesicles, a slight areola round one of them; all had a very active appearance, and the lymph taken was perfectly limpid and quite as adhesive as before. The decline of the vesicles on the eleventh day was perfectly obvious, and precisely as in the natural, casual, and innoculated vaccine. This was confirmed by the appearances on the twelfth day. On the twenty-sixth day, the crusts having fallen a day or two, the smooth pale rose-coloured scars were observed. Re-inoculation and re-vaccination here also proved unavailing. The lymph taken on the different days was used with different degrees of effect; but, when successful, produced perfect vaccine vesicles."

Mr. Ceely details at some length the experiment just mentioned. For the particulars we must refer to the paper itself, and content ourselves with adverting to some of Mr. Ceely's observations. "In the transfer," he says, "of the above lymph from the animals to man, my attention was forcibly arrested by the difficulties attending the process; and in the entire failure of so many punctures, the production of so many lymphless papulæ, and the formation of so few perfect vesicles, I recognized phenomena so common in similar trials with primary lymph. In some instances the difficulties were not completely overcome even in the second removes. The marked improvement, in subsequent removes, in the development of the vesicles, and the active manifestation of the primary and secondary symptoms, were not less apparent than in the use of natural lymph under corresponding circumstances, except that, in very few instances, and those principally in later removes and in peculiar subjects, there was not observed that disagreeable, inconvenient, and mischievous acrimony so peculiar to the former lymph. The lymph from the vaccine vesicles on the first experiment seemed to have acquired activity without causing the same amount of difficulty in its transmission.

These experiments with the variola vaccine lymph on man, show the necessity of having a number of subjects of different temperaments on which to employ it, on its first removes, to ensure success. It seems highly probable, too, that the direct transmission of the liquid lymph from the animal to children will save much trouble and conduce to greater success."

We scarcely think that any apology is due for the length at which we have considered the subject of vaccination. Its great intrinsic consequence—its particular importance at the present moment, when doubts are afloat regarding its efficiency—and, last not least, the satisfactory results of the late experiments, all plead our excuse, and, we trust, successfully. For the identity of the vaccine disease and variola may now be looked on as redeemed from the regions of conjecture, and the fears that existed for the deterioration of the vaccine lymph may now be fairly dispelled. It bears many removes, and at last, when its efficiency is failing, the cow may be confidently resorted to once more.

